# Prognosis and bio-psycho-social prognostic factors in children and adolescents with musculoskeletal pain consulting general practice

**DOI:** 10.1007/s00431-025-06217-2

**Published:** 2025-06-02

**Authors:** Negar Pourbordbari, Martin Bach Jensen, Jens Lykkegaard Olesen, Sinead Holden, Michael Skovdal Rathleff

**Affiliations:** 1 Center for General Practice at, Aalborg University, Selma Lagerløfs Vej 249, 9260 Gistrup, Denmark; 2 Department of Health Science and Technology, Faculty of Medicine, Aalborg University, Frederik Bajers Vej 7D, 9200 Aalborg, Denmark

**Keywords:** Children, Adolescence, Musculoskeletal pain, Prognosis, Prognostic factor

## Abstract

To investigate the 12-months prognosis and prognostic factors of 8-19-year-olds with musculoskeletal pain in general practice. A prospective cohort was conducted in a setting of Danish general practice clinics. Participants were children and adolescents aged 8–19 years old consulting the general practitioner with self-reported musculoskeletal pain completed a questionnaire at baseline, 3-, 6-, and 12-month follow-up. No interventions were applied. The primary outcome was activity-limiting pain at 6 months, regardless of pain location. We included one hundred children and adolescents (median age 13 years interquartile range 12–16.5, 55% female) with a follow-up rate of 70% at 6 months and 67% at 12 months. The most common pain site was the knee. After 6 months, 36% reported activity-limiting pain and 42% reported multi-site pain. After 12 months, 26% reported activity-limiting pain. Children and adolescents who felt nervous (odds ratio (OR) 4.2 95% confidence interval (CI) 1.4–12.5) or tired during the day (OR 2.9 95% CI 1.1–7.7), with 1–7 days of pain episodes (OR 7.1 95% CI 1.8–28.9), who used pain medication (OR 5.4 95% CI 1.6–18.4), had difficulties falling asleep (OR 4.8 95% CI 1.7–13.9), carrying a schoolbag (OR 3.8 95% CI 1.1–13.1), or bending down to put on socks due to pain (OR 4.1 95% CI 1.3–13.2) had a higher risk of pain after 6 months.

*Conclusion*: One-third of adolescents consulting their GP for MSK pain continue to experience activity-limiting pain at 6 months. This highlights the need for early identification of risk factors and a biopsychosocial approach to pain management in general practice. This study was performed at the Center for General Practice at Aalborg University, Fyrkildevej 7 1st floor, 9220 Aalborg Ø, Denmark. This work was supported by the Multidisciplinary Committee (MPU) providing a grant used for compensation for the general practice clinic’s time spent on recruitment [grant number ID: MPU 20–2017/date 100117]. ClinicalTrials.gov (Identifier NCT03678922) and link: https://clinicaltrials.gov/study/NCT03678922?term=NCT03678922&rank=1.

**Table:** 

**What is Known:**
• *Musculoskeletal pain, among other pain sites; headache and abdominal pain account for most of the recurrent painful states among children and adolescents; they may have a poorer prognosis and the MSK pain a larger impact than realized.*
• *Many adolescents consult their GP with MSK pain, but there is a lack of data in this setting of early stage management resulting in a limitation in the current body of evidence on the prognosis of adolescent MSK pain complaints is studies based on secondary care population or school-based population.*
**What is New:**
• *A substantial proportion of children and adolescents (36%) continue to experience activity-limiting MSK pain six months after consulting their GP, with 26% still affected after 12 months.*
• *Children and adolescents with persistent MSK pain at six months was characterized by functional difficulties in daily activities, all linked to their pain, - one of the most notable challenges was carrying a schoolbag.*

**Supplementary Information:**

The online version contains supplementary material available at 10.1007/s00431-025-06217-2.

## Introduction

Musculoskeletal (MSK) pain is highly prevalent in children and adolescents, affecting up to 69% of 8–16-year-olds within a school year [1]. One out of four adolescents between the ages of 10 and 15 years experiences MSK pain several days a week [2]. Six out of 10 experience pain in the lower extremities, followed by three in 10 who experience spinal pain [3]. MSK pain in youth is associated with reduced quality of life, school absenteeism, social withdrawal, anxiety, and sleep disturbance problems [4–8].

Each year, 8% of children and adolescents in the UK consult their general practitioner (GP) due to MSK conditions, with pain being the most frequent symptom [9]. While MSK pain often resolves, a substantial proportion develops persistent pain [10, 11]. Up to 36% of children transition from acute to chronic MSK pain [12] which significantly increases their risk of chronic pain in adulthood [13]. Chronic pain negatively impacts quality of life and increases the risk of psychological disturbances such as anxiety and depression [14]. Our recent systematic review of 26 studies (*n* = 16,241, aged 7–19 years) found 54–59% of adolescents still experience MSK pain after 1–9 years [11]. Musculoskeletal pain, among other pain sites, headache, and abdominal pain account for most of the recurrent painful states among children and adolescents [15]. Collectively, this highlights that adolescents with MSK complaints may have a poorer prognosis and larger impact than realized. Several biopsychosocial factors influence prognosis, including female sex, longer pain duration, fatigue, anxiety, multisite pain, and parental pain [11]. This is critical, since children exposed to early adverse life events might have a higher risk of cognitive, emotional, and health problems [16]. A limitation in the current body of evidence on the prognosis of adolescent MSK pain complaints is studies based on secondary care population or school-based populations. Many adolescents consult their GP, but there is a lack of data in this setting of early stage management [11]. This underscores the need to investigate the prognosis and prognostic factors in adolescents with MSK pain seen in general practice. This study aimed to describe the 3-, 6-, and 12-month prognosis of MSK pain in children and adolescents consulting general practice and identify candidate prognostic factors for persistent activity-limiting pain at 6 months.

## Materials and methods

This prospective cohort study followed the PROGRESS Prognostic Research Guidelines [17] and was reported using the STROBE guidelines [18]. It was an explorative prognostic study describing the course and outcome of MSK pain and identifying candidate prognostic factors for long-term MSK pain [19]. The Committee of Multipractice Studies in General Practice (MPU) approved the study (ID: MPU 20–2017, 100,117) [20], and it was waived by the North Denmark Region Ethics Committee (NVK, 090617) [21]. The study was registered on ClinicalTrials.gov (Identifier: NCT03678922). We refrained from stratifying the descriptive results by sex or by MSK pain type and from including a multivariable model due to a number of 100 participants [22].

We invited twenty-five GP clinics across Denmark to participate in the study, of which one clinic declined and twenty-four accepted. Seventeen clinics recruited participants for this study, and seven did not recruit any participants. Upon agreement of participation, participants completed a baseline questionnaire.

We included 8–19-year-old children and adolescents with self-reported MSK pain. MSK pain was defined as pain arising from muscle, tendon, bone, and joint as per the International Association for the Study of Pain (IASP) definition [23]. Inclusion criteria were as follows: (1) 8–19 years of age, (2) self-reported MSK pain (non-traumatic and traumatic caused by soft tissue damage, contusion or otherwise (excluding diagnosed fracture)), and (3) the ability to read and understand Danish/English. Exclusion criterion were as follows: Self-reported MSK pain known to be due to tumor, infection, or systemic (ex. rheumatic) and neurological causes.

The primary outcome was self-reported activity-limiting MSK pain at 6-month follow-up: musculoskeletal pain was considered a poor prognosis if participants reported pain in the past 2 weeks, leading them not to be able to participate in play in the school yard/spare time activities. Participants indicated pain site(s) on a predefined list. To ensure that responses reflected the impact of musculoskeletal symptoms, the questionnaire was introduced with a framing statement clarifying that children should answer the subsequent questions with reference to their experiences of pain. As such, responses regarding school absence were interpreted as related to the experience of pain rather than, for example, medical appointments. Participants could report headache (not considered a MSK pain site). Headache concurrently with neck pain is a previously identified prognostic factor for long-term neck pain. Headache without another concurrent MSK pain site would cause exclusion.

Participants were considered “recovered” at follow-up if they did not report activity-limiting pain at 6-month follow-up, regardless of pain site or if they reported other pain (not activity-limiting).

Prior to recruitment of the first included participant, the questionnaires were piloted [24]. We used a baseline questionnaire and a follow-up questionnaire administered at 3, 6, and 12 months (Supplementary Files 1 and 2). NP provided a link to the follow-up questionnaire to all participants at each follow-up timepoint. A text message was sent to participants who had not responded. Data was handled according to the Danish Data Protection Agency [25]. Our questionnaires were translated from English to Danish following the general methodology of translation, back-translation, and verification [13]. All 100 participants responded with the Danish questionnaire.

Due to our target audience of GPs, we wanted the terminology of our prognostic factors to be applicable in a GP setting. In order to gain recognition of this, NP created a temporary subgrouping, based on prognostic factors from our previous systematic review. She conveyed this subgrouping to a focus group [26] consisting of 15 clinically experienced GP physician peers.

Candidate prognostic factors are based on the literature and previous research [11] and on input from experienced clinicians during the development of the study (Tables 1, 2, and Supplementary Table). We measured body mass index (BMI), age, gender, and post code. In the scope of characterization of this study population, we thought inclusion of siblings and pubertal stage would be of interest. We included non-activity-limiting pain site(s). Multisite pain was based on the number of pain sites categorized as either activity-limiting or non-activity-limiting pain. If a participant had both, we used the number of activity-limiting pain sites. Pain episode duration at baseline was determined, and frequency of pain episodes and pain intensity was reported using the numeric rating scale (NRS) 0 to 10.

**Table 1:** Descriptive factors (demographics and pain characteristics) of study participants in the ChiBPS cohort at baseline (*N* = 100, *n* = %)

Demographics	
Age, median [IQR]	13 y [12–16.5]
Female sex, *n*	55
Siblings, *n*, median [IQR]	1 [1, 2]
Only child, *n*	7
Position in sibling line	
First	31
Second	36
Third/fourth	21
Youngest	41
BMI, kg/m^2^, mean	19.88 (4.86)
Pubertal stage, *n*	
Prepubertal	32
Pubertal	67
Pain characteristics	
Activity-limiting pain, *n*	
Knee	56
Ankle	18
Back	14
Non-activity-limiting pain, *n*	
Knee/neck pain	14
Back/ankle	10
Heel/foot	10
Pain duration, median [IQR]	5 mo [3 wk-1 y]
NRS, median [IQR]	7 [6–8]
Multi-site pain, *n* = 53	
2 sites	23
3 sites	14
4 sites	7
> 4 sites	9
Pain episode duration, *n*	
< 3 h	34
< 24 h	24
1–7 days	24
> 7 days	18
Pain episode frequency, *n*	
=/> Once/week	80
< Once/week	20
Radiculopathy, *n*	12

Data are based on 97%–100% replies. Position in sibling line, excluding only children and twins: fifth child, *n* = *3*, twins, *n* = *2*; pubertal status: one missing reply; multi-site pain: five participants reported only one pain site, and this was non-activity-limiting – as answer to pain question 3, of these one of the sites was the jaw. (ID 40, 42, 51, 57, 90); *IQR*, interquartile range; *NRS*, pain numerical rating scale; *y*, years, *mo*, months, *wk*, weeks

**Table 2:** Descriptive factors (psychosocial and physical activity characteristics) of study participants in the ChiBPS cohort at baseline (*N* = 100, *n* = %)

Psychosocial characteristics	
Pain outside school hours, *n*	97
Nervous, *n*	
Often/sometimes	34
Seldom/never	66
Worried or anxious, *n*	
Yes	33
No	32
I don’t know	35
Low self-esteem, *n*	
Yes	7
No	78
I don’t know	15
I believe in God, *n*	
Yes	36
No	35
I don’t know	29
Sleep per night, *n*	
</= 7 hs	22
8–10 h	75
10 h	3
Tired during the day, *n*	57
I have a job, *n*	33
I know the cause of pain, *n*	58
I expect the GP to prescribe pain medication, *n*	8
I expect a pain free near future, *n*	56
I expect a pain free long-term future, *n*	38
Pain affects my concentration, *n*	58
Pain medication,* n*	33
Frequency of pain medication, n	
Once/month	13
Once/week	12
> Once/week	6
Every day	1
Paracetamol, *n*	17
NSAID, *n*	9
Reason for consulting the general practitioner, *n*	
I want the pain to stop	57
I am worried for the cause of pain	53
My family made me come	22
I have a personal problem	2
I cannot use my body because of pain	63
Alcohol consumption, *n*	31
Cigarette smoking, *n*	3
Physical activity characteristics	
Physical active besides school hours, *n* = 80	
1 time/week	11
2–3 times/week	39
4–6 times/week	16
> 6 times/week:	5
Screen time/other activity mostly sitting down, *n*	
1–2 h/day	36
3–6 h/day	49
>/= 7 h/day	7
HFAQ, Pain makes it difficult to:, *n*	
Reach for a book on high shelf due to pain	10
Stand in a queue for 10 min	36
Carry my schoolbag to school	22
Sit on a chair for a 45-min lesson	31
Bend down to put on my socks	33
Sit up in bed after a lying position due to pain	5
Do sport activities at school	79
Run fast to catch a bus	67
Stand up from a lean chair due to pain	18
SDI, *n*:	
Difficult to fall asleep due to pain	38
Difficult to sit during a lesson	49
Pain disturbs a walk > 1 km	70
Pain disturbs physical exercise	88
Pain disturbs spare time activities	88

Data are based on 97%–100% replies; question concerning screen time had the lowest reply percentage. Pain medication: not mutual exclusive; physical activity: incl. one answer to: “sometimes once other times 3,” “1–2 times,” “1–3 times,” and “4–7 times,” two answers “3–4 times,” three answers: “3–5 times” and two answers to 0; screentime, outside school hours: excl. one answer of: “1–3 times,” “many times,” and “all the time,” and three answers of: “2–3 times”; *GP*, general practitioner; *NSAID*, nonsteroidal anti-inflammatory drug; *HFAQ*, the modified Hannover functionalability Questionnaire (more than one limitation could be ticked); *SDI*, Subjective disability index

Being worried or anxious, having low self-esteem, believing in God, feeling nervous, having expectations of a pain-free future, and having a job were determined. The cause of pain, pain outside school hours, and pain’s impact on concentration were determined. Reasons for consulting the GP, amount of sleep, alcohol, smoking, pain medication, and radiculopathy were also determined. The modified Hannover Functional Ability Questionnaire (HFAQ) [27] assessed limitations in 9 daily activities such as the following: reaching up to get a book from a high shelf, carrying a schoolbag to school, etc. The limitations were summed and categorized as low (0–1 limitation), moderate (2–3 limitations), or high (4–9 limitations). The subjective disability index (SDI) [28] was calculated from the answers to proposals such as the following: difficulties in falling asleep because of pain, or sitting during a lesson, etc. Previous research shows SDI 1–2 and 3–5 compared to 0 as a prognostic factors for long-term MSK pain [19, 27]. We included the limitations included in HFAQ and the proposals in SDI, due to their description of physical functioning limitations and clinical relevance.

### Statistical analysis

The proportion of knee, back, ankle, heel, and neck pain at all follow-up time points is presented among only those who had knee, back, ankle, heel, and neck pain respectively at baseline (Fig. 2). Data was exported from our REDCap questionnaires to an Excel table and checked for irregularities. Descriptive statistics were used to summarize data (Table 1 and Table 2). At all follow-up time points, the proportion of participants with activity-limiting pain was based on the proportion responding at that specific time point. Non-normally distributed data were described using median and interquartile range, and categorical data was described using percentages.

To identify candidate prognostic factors, an unfavorable outcome was defined as having activity-limiting pain at 6-month follow-up. Descriptive analysis was used to report the course of MSK pain over the 12-month follow-up period. We were interested in baseline measures associated with our outcome of pain at 6 months. We qualitatively summarized our candidate prognostic factors, focusing on a strong association based on the assumption of potential clinical relevance (defined in the authorgroup) as OR > 3 and 1 excluded from the 95% confidence interval. The analysis was presented with their central estimate and appropriate measure of dispersion (95% confidence interval). All prognostic factors are presented in association with pain at 6 months. The statistical analyses were conducted using STATA version 17.0. The event rates to some prognostic factors were low, and consequently, these items were either pooled or excluded from Fig. 3.^1^

## Results

**Fig. 1:**
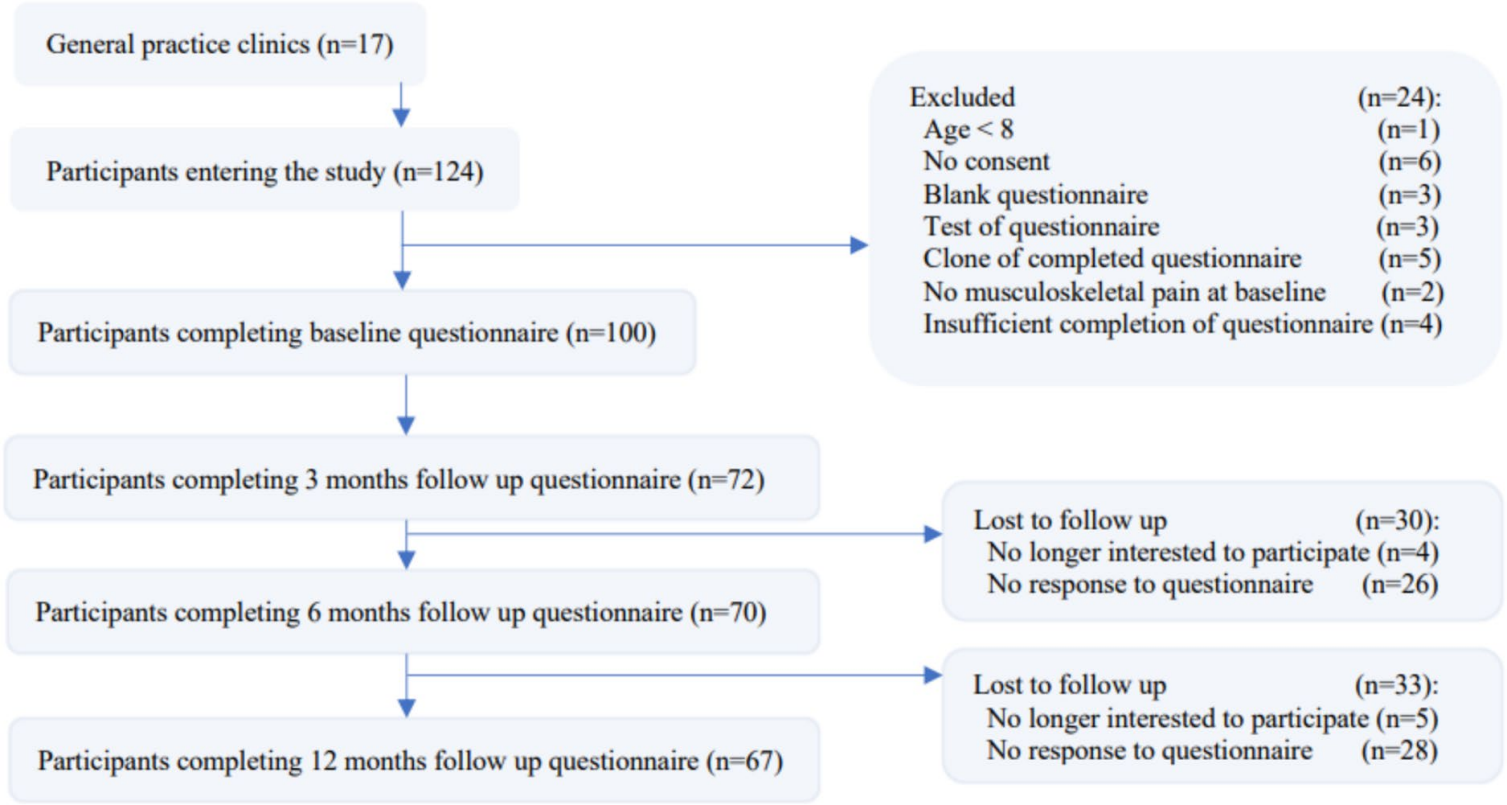
Flow chart

In total, 124 children and adolescents were identified from August 2018 to December 2020 from 17 GP clinics. Of these, 100 were included in the cohort (Fig. 1). There were no significant or relevant changes in baseline demographics or prognostic factors between responders and non-responders. The median age of participants was 13 IQR [12–16.5] years, and 55% of the cohort was female (Table 1). At baseline, 84% of the participants had activity-limiting pain, and 16% reported non-activity-limiting pain. Seventeen out of 100 participants reported not being able to attend school sometimes due to their pain (Fig. 3).

### Course and prognosis of MSK pain

**Fig. 2:**
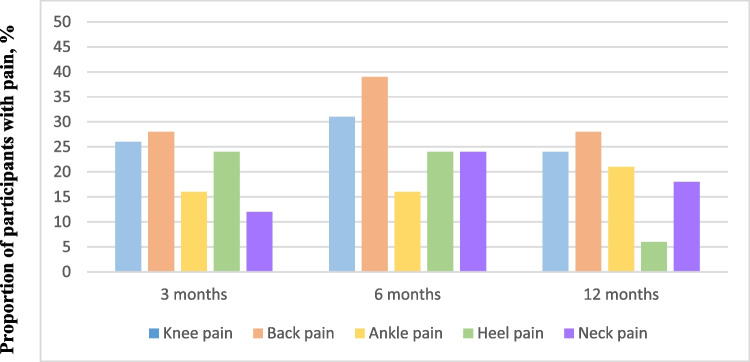
Prognosis of musculoskeletal pain. The proportion of 100 participants with baseline-activity-limiting MSK pain who had pain at 3, 6, and 12 months, stratified by pain site. The MSK pain depicted in Fig. 2 includes activity-limiting pain and nonactivity-limiting pain at the three time points. Bilateral pain, i.e., pain in two opposite body regions is considered mutually exclusive in the bars above. No mutual exclusivity for multi-site pain

All of the participants reporting activity-limiting pain at 6-month follow up reported activity-limiting pain at baseline. Figure 2 displays the five common MSK pain sites of our cohort. At 6-month follow-up, 36% of the participants had activity-limiting pain. Knee pain at baseline accounts for the majority of the 100 participants with a trajectory showing 27% with activity-limiting pain 3 months after inclusion and 32% with activity-limiting pain 6 months after. Twenty percent had activity-limiting pain 12 months after inclusion. At 12 months, 4% of those with baseline-activity-limiting knee pain reported non-activity-limiting pain. Response rates of the follow-up questionnaires were 72%, 70%, and 67% at 3-, 6-, and 12-month follow-up, respectively.

**Fig. 3:**
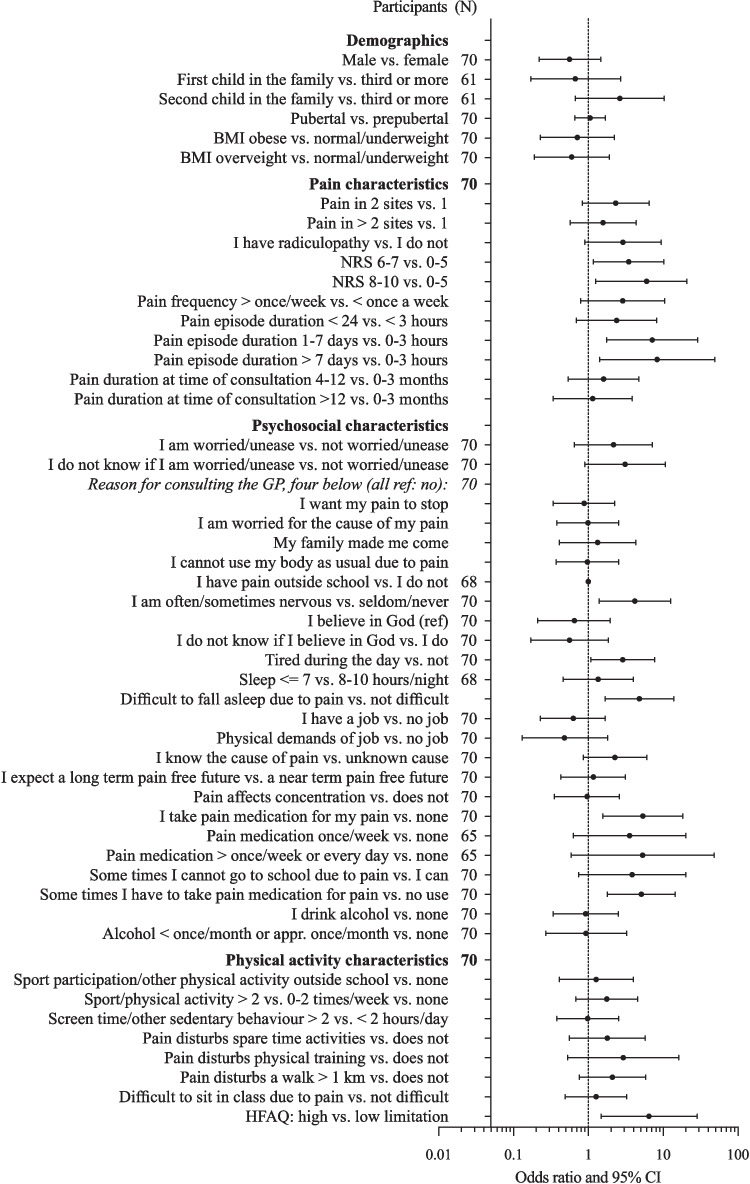
Activity-limiting musculoskeletal pain at 6 months. Odds ratio and 95% confidence intervals for prognostic factors of MSK pain at 6 months. *N* = participants in the statistical analysis. When *N* is stated next to a group of characteristics and not below in the listed characteristics, *N* is the same for all listed characteristics in this group

Figure 3 shows the ORs and 95% confidence intervals of all the potential prognostic factors. Data was based on the 70 children and adolescents who responded to the 6-month follow-up questionnaire.

Being the first or second child in the family, a higher BMI or a higher pubertal stage was not significantly associated with pain at 6-month follow-up. Ninety-six percent of the participants were born in Denmark.

A pain intensity of NRS 6–7 increased the risk of pain at 6-month follow-up (OR 3.5, CI 1.2–10.3) relative to NRS 0–5. A pain episode duration of 1–7 days increased the risk of pain at 6-month follow-up (OR 7.1, CI 1.8–28.9) relative to those with a pain duration of 0–3 h.

Difficulties with carrying a schoolbag due to pain increased the risk of pain at 6-months follow-up (OR 3.8, CI 1.1–13.1). Reporting difficulties in sitting for a 45-min class and standing in line for 10 min due to pain were almost clinically relevant (OR 2.6, CI 0.9–7.3) and (OR 2.8, CI 1.0–8.0). Difficulties with bending to put on socks increased the risk of pain at 6-month follow-up (OR 4.1, CI 1.3–13.2).

Feeling nervous often/sometimes increased the risk of pain at 6-month follow-up (OR 4.2, CI 1.4–12.5). Difficulties falling to sleep due to pain increased the risk of pain at 6-month follow-up (OR 4.8, CI 1.7–13.9), and feeling tired during the day was almost clinically relevant (OR 2.9, CI 1.1–7.7). Use of pain medication compared to no use was associated with 5.4 higher odds (1.6–18.4) of pain. Using pain medication sometimes increased the risk of pain at 6-month follow-up (OR 5.1, CI 1.8–14.4).

A higher HFAQ limitation increased the risk of pain at 6-month follow-up (OR 6.5, CI 1.5–28.3). Sport participation, amount of physical activity, and screen time did not have predictive value for pain at 6-month follow-up.

## Discussion

Our study highlights that a substantial proportion of children and adolescents (36%) continue to experience activity-limiting MSK pain six months after consulting their GP, with 26% still affected after 12 months. This persistence underscores the need to reconsider the management strategies of MSK pain in adolescents to improve outcomes.

The majority reported quite high pain levels and only 25% had a NRS of 6 or below. The group with persistent MSK pain at 6 months was characterized by functional difficulties in daily activities, all linked to their pain. One of the most notable challenges was carrying a schoolbag, a routine task for most children and adolescents; difficulty carrying a schoolbag due to pain was significantly associated with MSK pain persistence at six months. Previous research suggests that schoolbag carrying can serve as a proxy for physical strain and functional impairment in children with MSK pain [29–31]. However, a recent systematic review found no strong evidence linking schoolbag use to an increased risk of back pain in children and adolescents [32]. These mixed findings suggest that while schoolbag-related difficulties may indicate pain severity, they are unlikely to be a direct cause of MSK pain. Previous studies indicate that one in four schoolchildren with MSK pain use pain medication [2], whereas in our cohort, 42% of those with pain at 6 months used pain medication. While pain medication is often the first-line treatment and widely accessible [33], frequent use may indicate inadequate pain management or underlying pain severity rather than effective symptom relief. Additionally, we did not assess whether pain medication use was prescribed by the GP or self-administered, which could influence outcomes.

### Psychosocial characteristics and pain persistence

The children with knee pain reported more sleep problems and higher levels of nervousness compared to the children without pain. These findings resonate with previous studies demonstrating associations between pain, sleep difficulties, and emotional distress among adolescents [34, 35]. Poor sleep may exacerbate pain perception and hinder recovery, while nervousness may increase bodily vigilance and reduce engagement in physical activity, thus contributing to the persistence of symptoms. These interactions underscore the importance of addressing psychosocial dimensions when supporting children with knee pain. Considering that both sleep disturbances and psychological distress are potentially modifiable factors, early identification may help guide targeted interventions. Our findings highlight the need for adopting a biopsychosocial approach in primary care, where psychological, behavioral, and contextual factors are considered alongside physical symptoms when managing adolescent musculoskeletal pain.

### Pain characteristics and prognostic implications

Among the strongest pain-related prognostic factors for pain persistence at 6 months was pain episode duration exceeding 1 week. Our findings support the hypothesis that prolonged pain episodes (> 7 days) signal an increased risk of long-term pain persistence. Notably, while our study identified multiple strongly associated prognostic factors, the 95% confidence intervals (CIs) were wide, likely due to the small sample size and inherent variability. Some prognostic factors had CIs that included 1, indicating uncertainty in their predictive strength and whether they are associated with the prognosis. Future studies with larger sample sizes and formal hypothesis testing are needed to provide more definitive evidence on these associations.

### Comparison with existing literature

Previous research has identified female sex, longer pain duration, sleep disturbances, anxiety, and pain medication use as predictors of worse MSK pain prognosis [11]. Our findings align with these identified prognostic factors from these studies, but the setting of these are not GP clinics. The proportion of adolescents in our cohort reporting persistent pain at follow-up (approximately one in three) is in line with a recent systematic review which found a prevalence of chronic pain in children of 21%. This supports the generalizability of our findings and reinforces the clinical relevance of early identification and intervention in primary care [14]. Our findings furthermore suggest a potentially shorter pain duration at the time of GP consultation compared to other cohorts. This could indicate that patients in our study sought care earlier in their pain trajectory, potentially reflecting differences in healthcare-seeking behavior. Multi-site pain was highly prevalent in our cohort, with over half of participants reporting pain in multiple locations. This aligns with national findings indicating that one in three adolescents experience multi-site MSK pain [36]. Multi-site pain is a well-established risk factor for persistent pain in adulthood [37, 38], and our results further confirm its role in predicting long-term MSK pain persistence in adolescents [1, 28, 36, 39]. 

### Cognitive and functional impact of MSK pain

Difficulties concentrating have been frequently reported among schoolchildren with pain [2]. Our findings highlight concentration difficulties as a non-significant prognostic factor for MSK pain. Interestingly, some previously identified prognostic factors, such as shorter sleep duration (≤ 7 vs. 8–9 h per night) and occasional alcohol consumption, did not show significant associations with long-term MSK pain in our study. These discrepancies may be due to differences in sample characteristics, age distribution, and assessment methods.

### Strengths and limitations

One major strength of this study is a sample solely recruited from general practice. To our knowledge, this is the first study to explore the prognosis of adolescents with musculoskeletal pain consulting general practice. Another strength is that we differentiated MSK pain with a limitation on activity from otherwise pain. This is due to the commonality of pain and to distinguish the proportion of the MSK pain population reporting actual activity limitation due to pain. This distinction is important and ensures that we can differentiate pain with and without an impact on the individuals’ activity level that can vary from one child and adolescent to another. Candidate prognostic factors were selected on the basis of previous literature in our systematic review and clinical relevance. Self-report measures of pain and other factors impressionable by recall bias were limited by using a short recall period of 2 weeks.

It is unclear if our findings are generalizable to other countries with health care sectors, care-seeking behavior, and cultural differences than the Danish. Another limitation of this study, a small sample size led to uncertainty of the estimates and hindered a stratified analysis and multivariable model as originally planned. Furthermore, a limitation was response rates of 72%, 70%, and 67% at 3-, 6-, and 12-month follow up, respectively. A minor limitation is that participants either completed the baseline questionnaire before (32%), after the GP consultation (76%), or started before and completed after (11%) within days after the consultation. This variation may imply an increase or a decrease in reported expectations of pain medication and future pain duration, since the consultation (which separates these three possible answers) might have had an impact on answering questions related to expectations. It is unknown if adolescents reporting non-traumatic pain had a worse prognosis than adolescents reporting pain with a traumatic onset. We were not able to collect information on which treatments (if any) were prescribed by the GP.

## Conclusion

This study provides the first evidence-based insights into the prognosis of MSK pain among children and adolescents consulting general practice. Our findings highlight that one in four adolescents continue to experience activity-limiting pain even after 1 year. Identifying key risk factors, including longer pain duration, pain medication use, sleep disturbances, and psychosocial distress, may help GPs tailor early interventions to prevent long-term pain disability.

## Supplementary Information

Below is the link to the electronic supplementary material.

Supplementary Material 1 (DOCX 29.3 KB)

## Data Availability

No datasets were generated or analysed during the current study.

## Abbreviations

BMI: Body mass index
ChiBPS cohort: The Child and Adolescent Musculoskeletal Pain Cohort
CI: Confidence interval
GP: General practitioner
HFAQ: Hannover Functional Ability Questionnaire
IQR: Interquartile range
MPU: The Committee of Multipractice Studies in General Practice
MSK: Musculoskeletal
NP: Negar Pourbordbari
NRS: Numerical rating scale
NSAID: Non-steroidal anti-inflammatory drug
PDI-DV: The Pain Disability Index
OR: Odds ratio
SDI: Subjective disability index
STATA: Statistical software for data science
STROBE: Strengthening the Reporting of Observational Studies in Epidemiology

## Footnotes

1 We pooled the prognostic factors; ‘alcohol less than once/month’ with ‘alcohol approximately once/month’, ‘pain medication more than once/week’ with ‘pain medication every day’, and ‘job, mostly sedentary’ with ‘job, standing/walking’ because of their individual low event rates.‘Consulting the GP because of a personal problem’ (*n*=1), ‘cigarette smoking' (*n*=2), ‘sleep >10 hours vs. 8-10 hours/night’ (*n*=2), and reporting no to all nine limitations in HFAQ (*n*=1) were excluded because of low event rates.
