# Supplementary material for: Prognosis and bio-psycho-social prognostic factors in children and adolescents with musculoskeletal pain consulting general practice
Source: Eur J Pediatr. 2025 Jun 2;184(6):384. doi: 10.1007/s00431-025-06217-2 (PMC12129871; doi:10.1007/s00431-025-06217-2)
Supplement: Supplementary file 1 — Supplementary Material 1 (DOCX 29.3 KB) [file 431_2025_6217_MOESM1_ESM.docx]

**Supplementary Table, measurements**

This document is recommended to be read with the Supplementary File 1, Baseline questionnaire as it follows the same chronology. Measurements include those in the paper, here with reference to previous literature or GP focus group (when no other reference indicated). Previously identified prognostic factors with estimates (RR, OR (95% CI) or p-value) from our systematic review see Supplementary Table 1 [11]. Reference values are used for statistical analysis in calculating OR and 95% CI. Measurements are defined as descriptive (included in Table 1 and 2) or candidate prognostic factor (included in data analysis and Figure 3, unless otherwise stated).

| **Measurement** | **Possible answers** | **Categorization** | **Descriptive** | **Candidate prognostic factor** | **Reference used in statistical analysis, based on prev. res.** | **Previous prognostic factor** |
| --- | --- | --- | --- | --- | --- | --- |
| Sex | Boy, girl |  | x | x | Girl | [11] |
| Activity-limiting pain site(s) |  |  | x |  |  |  |
| Multi-site (activity limiting pain) yes/no |  |  | x | x |  | [11] |
| Non-activity-limiting pain site(s) |  |  | x |  |  |  |
| Pain can easily be ignored | Yes, no |  |  | x | No |  |
| Pain affects my concentration | Yes, no |  | x | x | No |  |
| Sometimes I have to take pain medication due to pain | Yes, no |  |  | x | No |  |
| Sometimes I can’t attend school because of pain | Yes, no |  |  | x | No |  |
| NRS | Open answer, 0 to 10. | NRS 1-5: mild interference with functioning, 6-7: moderate interference, and 8-10 severe interference with functioning^nrs^ [11]. | x | x | NRS 1-5 | More intense pain on VAS 0-10, p=0.03 [11]. |
| Pain duration at baseline | Open answer | 0-3 months, 4-12 months, and > 12 months | x | x | 0-3 months | Longer disease duration before first admission, p<0.01 [11]. |
| Known cause of pain^branch^ | Yes, no |  | x | x | No |  |
| Pain episode duration, exp^a^ | < 3 hours, < 24 hours, 1-7 days, and > 7 days |  | x | x | < 3 hours | Prognostic for low back pain and back pain [40,41]. |
| Pain episode frequency, exp | More than once a week, less than once a week |  | x | x | Less than once a week | [4,5,40] |
| Expectations of a pain free future | Near future, long term future, no |  | x | x | Near future |  |
| Pain medication | No, yes and I know the name of the pain medication, yes but I do not know the name |  | x | x | No |  |
| Frequency of pain medication | Once a month, once a week, several times a week, every day |  | x | x | No pain medication in the previous question. |  |
| Radiculopathy^a^ | Yes, no |  | x | x | No | Prognostic factor for low back pain [11]. |
| Reason for consulting the doctor | Multiple choice (see Suppl. File 1). |  | x | x | No |  |
| Expectations for pain medication | Yes, no |  | x | x | No |  |
| First visit to the GP concerning current MSK condition^branch, notana^ | Yes, no |  |  |  | No |  |
| Worried and anxious^exp^ | Yes, no, I do not know |  | x | x | No |  |
| Low self-esteem^exp^ | Yes, no, I do not know |  | x | x | No | [11] |
| God | Yes, no, I do not know. |  | x | x | Yes | Religious people are less likely to have chronic pain and tiredness [42]. |
| SDI, exp | Five proposals. Multiple choice (see Suppl. File 1) |  | The individual proposals are each defined as descriptive factors. | The SDI score based on the sum of all proposals is defined a candidate prognostic factor. | Score of zero | SDI 1-2 and 3-5 compared to 0 is a prognostic factor for long term MSK pain [11]. |
| Pain outside school hours | Yes, no |  | x | x | No |  |
| Tired during the day, exp | Yes, no |  | x | x | No | [37] |
| HFAQ, exp^a^ | Nine limitations. Multiple choice (see Suppl. File 1) |  | The individual limitations are each defined as descriptive factors and not as candidate prognostic factors. | The HFAQ score, based on sum of limitations is defined as a candidate prognostic factor. | Score of zero | A high HFAQ score compared to a low score has previously been described as a prognostic factor for persistent pain among participants with low back pain when asked about limitations caused by pain [27,40]. |
| Screen time^a^ | Open answer | 0-2 hours a day, > 2 hours a day. | x | x | 0-2 hours a day | We used a cut off value of 2 hours a day in the analysis with reference to differences in the proportion of schoolchildren using screens more than two hours and reporting low back pain during the week - compared to those who reported using screens less than two hours [43]. (These differences were not observed on weekends). |
| Sleep | 7 hours or less, 8-10 hours, more than 10 hours |  | x | x | 8-10 hours | An u shaped relationship has been observed between length of sleep duration and MSK pain. </= 5 hours and >/= 9 hours sleep duration are both significantly associated with MSK pain among adults in comparison to 7 hours sleep [37]. We use the same cut off limits on our child and adolescent population, as a moderated reference and categorize in: 7 hours or less, 8-10 hours, more than 10 hours sleep. |
| Physical active beside school hours | Yes, no |  | x | x | No |  |
| Frequency of sport participation | Open answer | Post data collection dichotomization in: 0-2 times a week, > 2 times a week |  | x | 0-2 times a week | [11] |
| Alcohol | Yes, no |  | x | x | No |  |
| Cigarette | Yes, no |  | x | x | No |  |
| Job | Have a job, does not have a job. |  |  | x | Does not have a job |  |
| Nervous, exp | Seldom/never, often/sometimes |  | x | x | Seldom/never | [41] |
| Zip code^notana^ | Open answer |  | x |  |  |  |
| Country of birth^notana^ | Denmark, other country |  | x |  |  |  |
| Years lived in Denmark^notana^ | Open answer |  | x |  |  |  |
| Siblings | I don’t have any siblings, 1, 2, 3, 4, 5, 6, more than 6 | At least the third sibling in the row of siblings, number two child in the row of siblings, first child. | x | x | Being the third child or more, in the row of siblings is the least prevalent in our ChiBPS cohort of children and adolescents with MSK pain. We used this group from our categorization as our reference in the statistical analysis. |  |
| What nationality do you feel the most as?^notana^ | See Suppl. File 1 |  | x |  |  |  |
| Friends with immigrant background?^notana^ | None, almost none, almost all, all |  | x |  |  |  |
| Pubertal stage^tan^ | Tanner 1 to 5 | To investigate the association of pubertal stage to the persistence of MSK pain, we stratified our cohort in two groups: 1) prepubertal 2) pubertal at baseline [45]. | x | x | Tanner 1-2 | Previous research show that MSK persists significantly more in higher pubertal groups rather than the first group (the highest group being the most significant), which we base our categorization of pubertal stages on [44]. |
| BMI^bmi^ | Weight and height separately open answer | Normal/underweight < 18.35 kg/m^2^, overweight 18.35-21.57 kg/m^2^, obese > 21.57 kg/m^2^ | x | x | Normal/  underweight | Adolescent knee and hip pain are associated with increased BMI (overweight and obese) [45]. |
| Time of consultation with pain^notana^ | See S1 File |  | x |  |  |  |
| Time of completion of questionnaire^notana^ | See S1 File |  | x |  |  |  |

NRS (numeric rating scale): The optimal cut-off points for mild, moderate, and severe pain terms of pain-related interference with functioning (assessed with the pain disability index (PDI-DV), a 7-item questionnaire to investigate the magnitude of self-reported disability in different situations such as work, leisure time, self-care, and social activities) in patients with chronic MSK pain.

Branch: if answered yes, branching to open answer of cause of pain, name of pain medication or number of visit to the GP. Cause of pain and the number of the current visit not included in our analysis. Name of pain medication included when a non-steroidal anti-inflammatory drug (NSAID). Alcohol: Followed by question of frequency of alcohol with five possible answers. Frequency also defined as candidate prognostic factor. Cigarette: Followed by question of frequency of alcohol with four possible answers. Frequency also defined as candidate prognostic factor. Job: Followed by question of physical activity level at work with five possible answer. Descriptive and candidate prognostic factors. Siblings: Followed by number in the row of siblings. Open answer question. This also a descriptive as well as a candidate prognostic factor.

Exp: exposures included in our protocol. Anxious an exposure, but worried was not.

a: Previous prognostic factors for low back (radiculopathy, HFAQ and screen time) and back pain (pain episode duration). Included here as a candidate prognostic factor for general MSK pain, because of a cohort of no more than 100 participants. HFAQ: We predicted a prognostic value of a *modified* HFAQ score among our MSK pain participants.

Notana: not included in the statistical analysis.

Tan: Previous research defines pubertal stage 3 as Tanner 3-5 and pubertal stage 2 as adolescents who became pubertal during a 3 year follow up period [44]. Since we do not have the same study duration we instead use the cut off value: Tanner 3, in calculating significance.

BMI (body mass index): To report BMI as a measure, we used international cut off points for overweight and obesity by age 8-18 years, obtained in previous research by averaging the centile curves for overweight by age 0-20 [46].
